# Adachi Child Health Impact of Living Difficulty (A-CHILD) Study: Research Protocol and Profiles of Participants

**DOI:** 10.2188/jea.JE20190177

**Published:** 2021-01-05

**Authors:** Manami Ochi, Aya Isumi, Tsuguhiko Kato, Satomi Doi, Takeo Fujiwara

**Affiliations:** 1 Department of Health and Welfare Services, National Institute of Public Health, Saitama, Japan; 2 Department of Social Medicine, National Research Institute for Child Health and Development, Tokyo, Japan; 3 Department of Global Health Promotion, Tokyo Medical and Dental University, Tokyo, Japan; 4 Japan Society for the Promotion of Science, Tokyo, Japan

**Keywords:** child in poverty, child health, life course, longitudinal study, Japan

## Abstract

**Background:**

The Adachi Child Health Impact of Living Difficulty (A-CHILD) study has been conducted since 2015 to clarify the associations between socioeconomic factors and child health, as well as to accumulate data for political evaluation of the child-poverty agenda. This paper describes the purpose and research design of the A-CHILD study and the baseline profiles of participants, together with the future framework for implementing this cohort study.

**Methods:**

We have conducted two types of continuous survey: a complete-sample survey started in 2015 as a first wave study to target first-grade children in all public elementary schools in Adachi City, Tokyo, and a biennial fixed grade observation survey started in 2016 in selected elementary and junior high schools. Questionnaires were answered by caregivers of all targeted children and also by the children themselves for those in the fourth grade and higher. The data of A-CHILD also combined information obtained from school health checkups of all school-grade children, as well as the results from blood test and measurement of blood pressure of eight-grade children since 2016.

**Results:**

The valid responses in the first wave were 4,291 (80.1%). The number of households in “living difficulties”, such as low household income or material deprivation, stood at 1,047 (24.5%).

**Conclusions:**

The A-CHILD study will contribute to the clarification of the impact of poverty on children’s health disparities and paves the way to managing this issue in the community.

## BACKGROUND AND PURPOSE

### Child poverty in Japan

In Japan, child poverty has recently become a major political issue. A United Nations Children Fund study showed that the financial and material deprivation level of Japanese children was relatively higher than that of children in other developed countries.^1^ The proportion of children who lived in households below the poverty line, that is, households with less than half of the median household-size-adjusted income of the population, was 16.3% as of 2012,^2^ rendering Japan to be ranked 12^th^ among 35 Organisation for Economic Co-operation and Development countries.^3^ Confronted with child poverty in Japan, an act to accelerate policies for disadvantaged children was implemented in 2013, with all levels of local governments to tackle the various problems due to child’s poverty. In such circumstances, while many related literature reviews and policy descriptions were published in Japan, there is a lack of studies which use quantitative data to study the effects of the poverty on child health and development. Moreover, in spite of the presence of longitudinal studies in some developed countries,^4^^,^^5^ such well-designed studies are still scarce in the Japanese context.^6^ Cross-sectional study about recent conditions among children does not enough to help our knowledge about developmental processes that could be a clue for the prevention and early intervention efforts. Therefore, it is of practical and academic value to conduct a longitudinal study that not only studies the effect of poverty on child health and development in Japan, but also evaluates each policy for disadvantaged children in poverty.

We herein describe the aim, conceptual content, study design, and the characteristics at baseline and following waves of a cohort study, the Adachi Child Health Impact of Living Difficulty (A-CHILD), which started in 2015 with the purpose of monitoring and revealing factors related to the health and development of the child in poverty in Japan.

### Project for child poverty in Adachi City

Adachi City is one of the 23 special wards in Tokyo Prefecture, and is located in northernmost area of Tokyo. As of 2015, the population in Adachi City was approximately 690,000. The average income in Adachi City is 3.3 million JPY (1 USD ≈ 122 JPY) in 2015,^7^ which is similar to the average in Japan (3.1 million JPY) but lower than that of the whole Tokyo (4.5 million JPY).^8^ The proportion of population that received public aid was 3.7% in 2015, which was the highest rate in Tokyo Prefecture (mean 2.2%). The healthy life expectancy in Adachi City is 2 years shorter than that of Tokyo Prefecture,^9^ which suggests a health disparity between the people in Adachi City and those in other areas in Tokyo. One possible reason for this could be that there are more people in Adachi City who suffered from lifestyle chronic diseases, such as diabetes complicated with kidney diseases,^9^ which could have stemmed from poor dietary habits or overweight in childhood. It has been shown that the origins of adult health disparities were consequential of the physical and developmental damages due to poverty in the early periods of childhood.^10^^,^^11^

Under the leadership of the mayor of Adachi City, Yayoi Kondo, the city government initiated the “Adachi Project Connected to the Future (child poverty implementation plan of Adachi City)” in fiscal year 2015 with aim of enabling children to adopt a desirable lifestyle and enjoy good health without being affected by household conditions or the environment where they were born and raised in. The political framework and goals for disadvantaged children were viewed by the Japanese Cabinet Office as a model case by a local government.^12^ In the same year, in collaboration with the Adachi City government, we started A-CHILD study. Before the “Adachi Project Connected to the Future” was disseminated, we were able to obtain the data of the first wave, which would show the baseline status of children and their families. We also follow up with targeted children for the implementation period of the project in Adachi City.

### The overall concepts and aims of A-CHILD study

A sizeable number of studies in medicine, psychology, and social epidemiology have demonstrated that early-childhood experiences of poverty have extended impacts on their lifelong physical and psychological development.^13^^–^^16^ Childhood poverty is also related to an increased risk of poor academic achievement, which may consequently lead to lower income,^5^^,^^17^ resulting in “the cycle of poverty” through generations.

Recent studies about child poverty have focused on not only monetary but also multidimensional aspects of poverty,^18^^–^^21^ whose evidence would provide a broad understanding of the actual deprivations with which children are confronted. Monetary poverty indicators, such as low household income, have been widely used for studies of child poverty; however, monetary indicators alone would not be sufficient to capture the state of poverty because of the lack of capturing specific values, such as properties, debts, and benefits in-kind.^22^^,^^23^ Therefore, alternative multidimensional poverty approaches have been developed,^21^^,^^24^ including Townsend’s relative deprivation,^25^ basic needs,^26^ or the social exclusion approach.^19^^,^^27^ By viewing child poverty from multiple perspectives, it will be possible to examine the path that poverty could have on children over the long term. But so far in Japan, few studies have identified both multiple aspects of poverty and child’s health outcomes.

Based on the recent policy and research situation regarding child’s poverty in Japan, our aims of A-CHILD study are as follows. First, we describe the current situation of children in poverty and their health status in a local-governmental area. In collaboration with the Adachi City in Tokyo, we conducted a longitudinal study targeting children and their families from the fiscal year that this city had started taking action for disadvantaged children. Second, we focus on the effect of not only the monetary aspects but also the multidimensional aspects of child poverty to explore the impact of low income, material deprivation, or payment difficulty in family on the child’s health and development. We will examine some specific concerns, for example, whether material deprivation, such as the lack of appropriate books/toys for children’s age, would affect children’s psychological development, even if children’s household is not low-income. Third, we aim to reveal the mechanisms and impact of local governmental policies on the reduction of health disparities in children. For example, food education emphasizing eating vegetables has been implemented at a public nursery school in Adachi City. Therefore, we will investigate whether the dietary habits cultivated before attending school would affect the dietary habits and physical growth of children after schooling, regardless of the family’s economic situation. An important aim of the A-CHILD study is to elucidate the modifiable mediator of the link between poverty and child’s health, which would break the cycle of intergenerational poverty.

## PARTICIPANTS AND FOLLOW-UP

### Study design

A-CHILD study is broadly divided into “original survey” using questionnaires developed for A-CHILD study itself, and another part using data of the survey and medical examination that Adachi City has carried out for elementary and junior high school students. The original survey also included two types of surveys: longitudinal surveys that began in the first grade of elementary school, and biennial cross-sectional surveys of fourth grade or older. “Wave” indicates the year of the survey since the first year of A-CHILD study. The details and purpose of each survey are shown below.

### Original questionnaire survey

**Figure 1.**
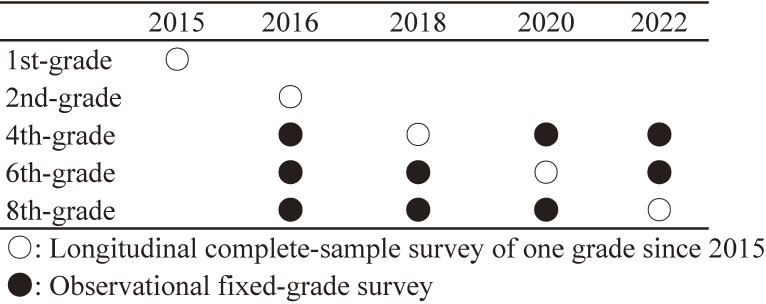
Target grade and survey schedule in A-CHILD

We have conducted two types of continuous survey. The first was a complete-sample survey started in 2015 as a first wave study to target first-grade children (aged 6–7 years old) in all public elementary schools in Adachi City. In order to create the panel data that was started from the first grade, we conducted a follow-up survey on the children who participated in the first wave (Figure 1). Questionnaires were answered by caregivers of all targeted children. After completing the questionnaire, the children returned it at school in an anonymous sealed envelope. We did a pilot to test processes at six elementary schools in July 2015. Although minor changes were made to the procedure on the survey after the pilot, there were no major changes to the content of the questionnaire. The main survey was conducted in the remaining 63 elementary schools in November 2015.

From the second wave, we conducted a survey every other year since November 2016 to target the children in fourth and sixth grades in elementary school (aged 9–10 and aged 11–12 years, respectively) and second grade in junior high school (ie, eighth grade, aged 13–14 years) (Figure 1). This second type of survey enabled us to examine changes over time in each grade by investigating the same grades repeatedly in different years. The questionnaire was answered by caregivers and their children. Adachi City is divided into five administrative areas, each with different geographical characteristics. Therefore, we asked for cooperation in the survey so that we could get answers from at least one elementary and a junior high school from each administrative area. As a result, nine elementary schools and seven junior high schools were selected, which in major urban areas with hub stations or in residential areas away from the station. That is, the second wave comprised both a complete sample survey of second-grade children in all elementary schools and surveys targeting some children in the fourth, sixth, and eighth grades. Participants were informed that participation in the study was voluntary and that participants could indicate in the questionnaire their disagreement if when they did not agree to participate in the survey. All children were assigned a unique ID by an administrative officer of Adachi City, and we can access only the data without personal information, such as a child’s name and class number. Since the second and subsequent waves, the annual data of each survey were linked to this ID.

### Data from Study Attitude survey and school checkups

In the second wave in 2016, we also used data from the Study Attitude survey, which was conducted in the same fiscal year by the Board of Education of Adachi City. The Study Attitude survey is one about the attitudes and lifestyles related to learning of elementary and junior high school students in Adachi City. We were provided the Study Attitude survey data of the same grade with the original questionnaire survey (ie the second-, fourth-, sixth-, and eighth-grade children). Because some questions in the Study Attitude survey were the same within the original questionnaire survey in the first wave of A-CHILD, the original questionnaire since the second wave avoided asking the same questions as the Study Attitude survey to reduce the burden on the children.

The data of the A-CHILD study features objective child health information obtained from mandatory school health checkups including physical measurement and dental checkup. Further, with caregiver’s consent, the eighth-grade children were invited to undergo a “child lifestyle-related health checkup”, which included a blood test and the measurement of blood pressure.

**Figure 2.**
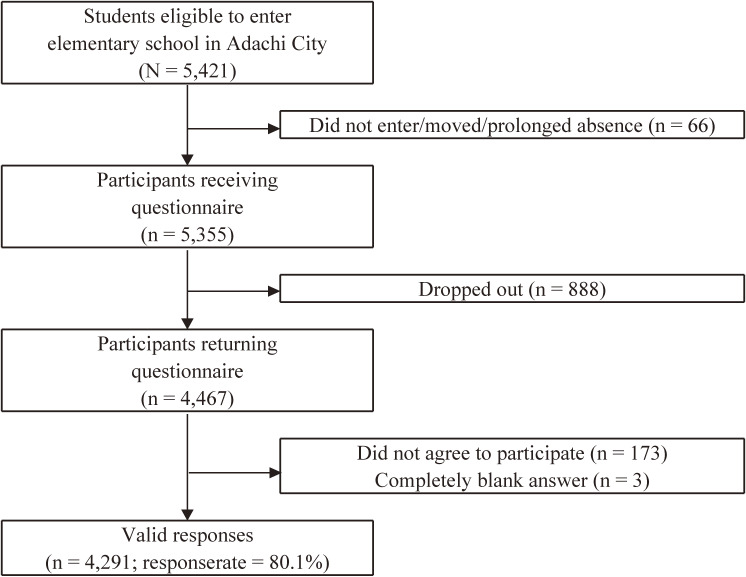
Flowchart of analytical sample of A-CHILD in first wave in 2015

**Figure 3.**
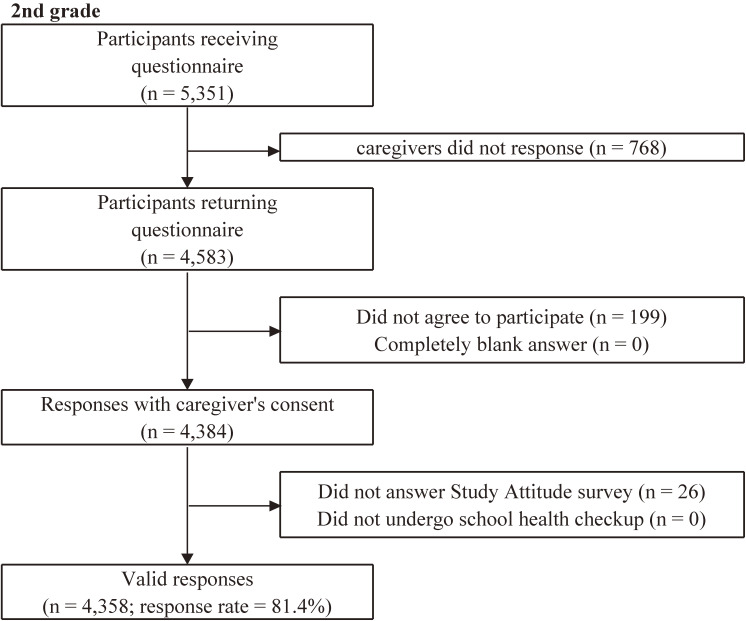

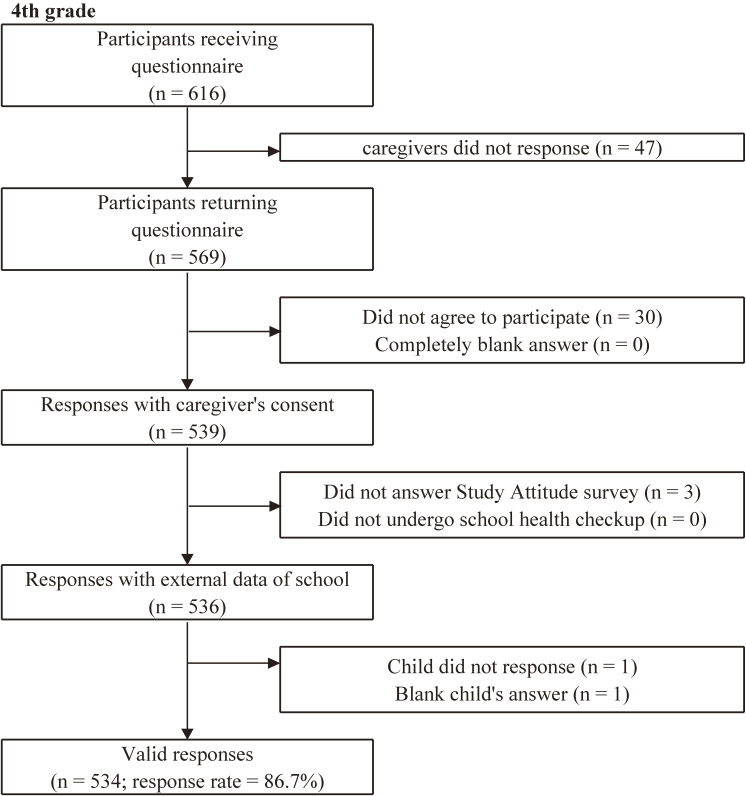

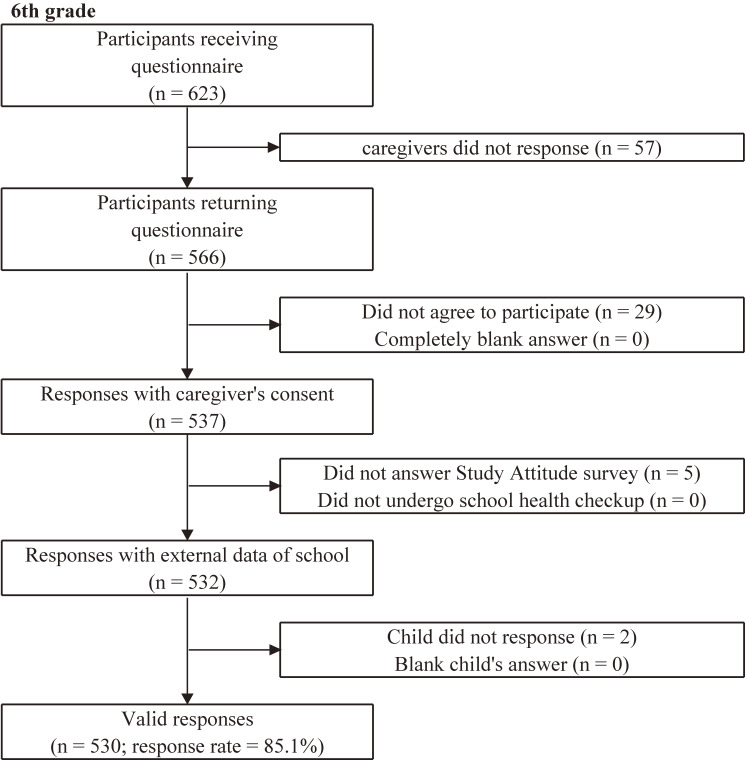

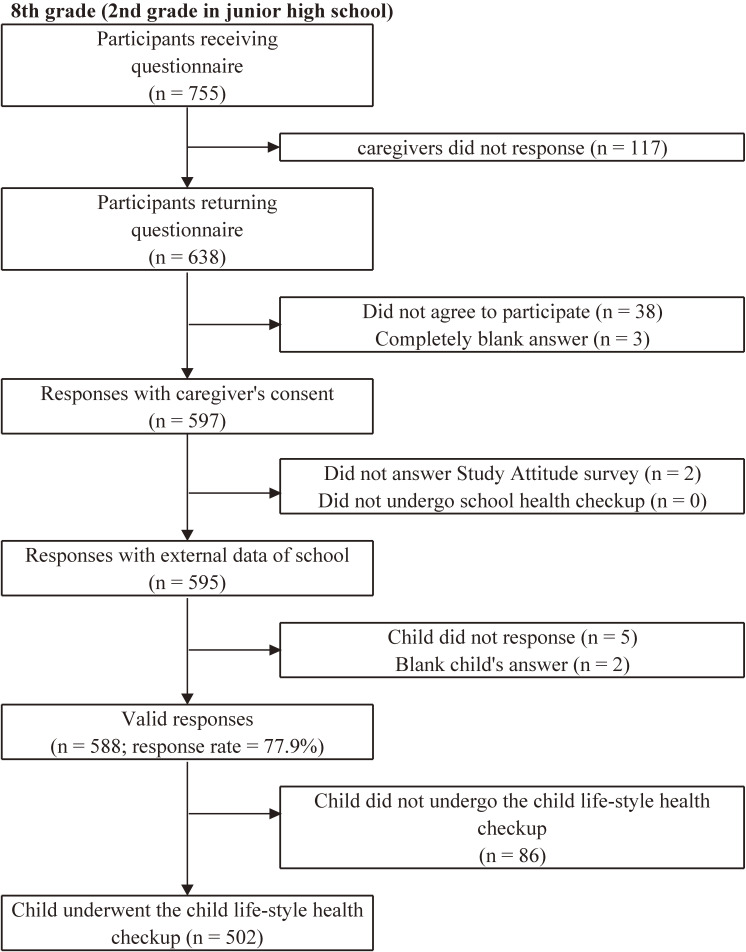
Flowchart of the analytical sample of A-CHILD in the second wave in 2016

Among the original questionnaires returned, we adopted as valid responses only the answers that could be merged with the data of the Study Attitude survey and school health checkup via a unique ID, excluding the questionnaires from caregivers who did not agree to participate or those that were submitted blank. The questionnaire answered by the child was validated only when the child him/herself agreed to participate, and the caregivers also agreed that their children answered. The flowcharts of the participant recruitment are shown in Figure 1, Figure 2, and Figure 3. We received the endorsement for each survey from the Adachi City government, which contributed credibility and higher participation rates of this study.

### Main outcome measures

We prepared a set of questionnaire items relevant to the circumstances of poverty and other social determinants of health and those underlying mechanisms based on currently available theories. In elaborating the questionnaire, the research team referred to the opinions of Adachi City administrative officers from the viewpoint of implementation at schools.

Table 1 summarizes the measures in the first wave questionnaire answered by respondents (ie, caregivers): (1) household demographic factors; (2) demographic factors of children; (3) parental characteristics, which were asked about both mother and father of the child if available; (4) parenting factors, which included parental involvement with children and child maltreatment; (5) respondent’s psychosocial characteristics; (6) child’s lifestyles and behavior. Details of the scales for variables are described in the online supplementary material.

**Table 1.** List of measures answered by caregivers in A-CHILD study in 2015 and 2016

	1st grade in 2015 (wave 1)	2nd grade in 2016 (wave 2)	4th, 6th, and 8th grade in 2016 (wave 2)
**Demographics of household**			
Families living in the same house	○	○	○
Families living away from home for work	○		○
Parental marital status	○		○
Changes in parents’ marital status in the last year		○	
Frequency of cooking at home	○	○	○
Frequency of eating vegetables	○	○	○
Annual income in the last year	○	○	○
Receipt of public aid and pension	○	○	○
Material deprivation	○	○	○
Payment difficulty	○	○	○
**Demographics of children**			
Sex	○		○
Birth date	○		○
Pre-school facilities	○		○
Weight and gestational week at birth	○		○
Weight and height at 3 years old	○		○
Vaccination history	○		○
Experience of hospitalization	○		
Hospitalization in the last year			○
**Parental characteristics**			
Age	○		○
Height	○		○
Weight	○		○
Smoking habit	○		
Alcohol consumption	○		
Physical activity	○		
Diagnosed disease history	○		○
Education	○		○
Habit of reading books	○		○
Working status	○	○	○
Time of returning home	○	○	○
**Parenting**			
Parental involvement with children	○	○	○
Child maltreatment	○	○	○
Parenting when children claim hungry	○		
Visiting dental clinic if children diagnosis caries		○	○
Source of information on child health services		○	○
**Respondents’ psychosocial characteristics**			
Relationship with children	○		
Happiness	○	○	○
Mental health (K6)	○	○	○
Years of residence at present address	○	○	○
Neighbourhood social capital	○	○	○
Social network	○	○	○
Belonging community groups		○	○
Eating habit of starting meal		○	○
Self-rated health		○	○
**Lifestyles and behavior of children**			
School refusal	○	○	○
Dietary habit of breakfast	○	○	
Dietary habit of school lunch	○	○	
Dietary habit of dinner	○	○	
Dietary habit of sweets and sugary drink	○		
Types of food children eat first at meal	○	○	
Dietary habit of sweets and sugary drink		○	
Toothbrushing habit	○	○	○
Number of caries	○	○	
Wake-up and bedtime habit	○		
Physical activity	○	○	
Sports club activity		○	
Frequency of leaving at home alone	○	○	
Habit of watching TV or video	○		
Habit of playing computer game	○		
Habit of reading books	○		
Places to spend after school		○	
Participation for events in the community		○	○
School social capital		○	
Resilience and coping behavior (CRCS)	○	○	○
Behavior problem and prosocial behavior (SDQ)	○	○	○

CRCS, the Children’s Resilient Coping Scale; SDQ, the Strengths and Difficulties Questionnaire.

The actual questionnaires in A-CHILD surveys are available online in Japanese; http://www.city.adachi.tokyo.jp/kokoro/fukushi-kenko/kenko/documents/adachi-shitsumon271124.pdf (questionnaire in wave 1), https://www.city.adachi.tokyo.jp/documents/30759/4shiryou.pdf (questionnaire in wave 2).

The question items in the second wave answered by the caregivers of the second-grade elementary school children are almost the same as those in the first wave. However, some of the questions about development in early childhood and parental characteristics were excluded from the question items because these data had already been acquired in the first wave. On the other hand, questions were added to the second wave items to obtain more details than those in the first wave, such as parents’ membership of community groups and children’s participation for events in the community (Table 1).

The question items for the caregivers of the children in fourth, sixth, and eighth grades in the second wave were almost the same as those for caregivers of first-grade children in the first wave, but did not include the same items in the questionnaire for children who would be responding themselves. We also modified several sentences in the questionnaire to capture what we would like to know based on the qualitative and quantitative review of results in the first wave.

Table 2 summarizes the items answered by children since the second wave. These items included children’s lifestyles, such as eating habits, wake-up time and bedtime, toothbrushing, frequency of reading books, and places they spent time after school. It also included subjective questionnaires, such as school social capital and their self-esteem. According to child’s grades, we also added questions on whether they could do simple cooking on their own (for the children in sixth and eighth grades only) and whether they were interested in dieting (for children in the eighth grade only).

**Table 2.** List of measures answered by children in A-CHILD study in 2015 and 2016

Measures answered by students of 4th, 6th and 8th grade students in the second wave survey in 2016		
**Question items in A-CHILD questionnaire**		
Dietary habit of breakfast	Sports club activity	Coping behavior
Dietary habit of dinner	Physical activity	Existence of adults other than parents
Types of food children eat first at meal	Time spent on mobile devices	Happiness
Dietary habit of sweets and sugary drink	Places to spend after school	Self-esteem (subscale of Sakurai et al)
Wake-up and bedtime habit	School social capital	Self-cooking ability (6th and 8th grade students only)
Time to take a bath	Social network among children	Interest in dieting (8th grade students only)
**Question items in Study Attitude survey**		
Habit of reading books	Toothbrushing habit	Habit of playing computer game
Dietary habit of school lunch	Habit of watching TV or video	Habit of using the Internet

Measures in school health checkup of A-CHILD survey in 2015 and in 2016		
**Physical measurement**		
Birth date	Height	Weight
**Dental checkup**		
Diagnosis on deciduous teeth (with position in 2016)	Diagnosis on permanent teeth (with position in 2016)	Other findings (malocclusion, temporomandibular joint, plaque, gums)
**Blood test (parts of 8th grade students only)**		
Blood pressure	LDL-cholesterol	Hematocrit (Ht)
Total cholesterol (TC)	Red blood cells count (RBC)	Hemoglobin A1c (HbA1c)
HDL-cholesterol (HDL-C)	Hemoglobin (Hb)	Glycosuria

The actual questionnaires in A-CHILD surveys are available online in Japanese; https://www.city.adachi.tokyo.jp/documents/30759/4shiryou.pdf (questionnaire in wave 2).

The questionnaires in Study Attitude survey in Japanese conducted by Adachi City are available if required.

Physical measurement and dental checkup in the school health checkup were assessed according to a national-standardized guideline.^28^ Details of how each checkup was conducted are described in the online supplementary material.

This study was approved by the Ethics Committee of the National Center for Child Health and Development (approval number: 1147) and Tokyo Medical and Dental University (approval number: M2016-284). All analyses were performed using the computer software STATA14 for Windows (STATA Corp., College Station, TX, USA).

## BASELINE DESCRIPTIVE STATISTICS

### The first wave (pilot survey & main survey)

We identified 5,355 children who would enter elementary schools in Adachi City and were eligible for the first survey. Among the questionnaires distributed, 4,467 were returned. We excluded questionnaires that were left blank, which yielded 4,291 valid responses (80.1%). The selected characteristics of the first wave are shown in Table 3.

**Table 3.** Selected profiles of the participants for the 1st wave of A-CHILD study

	1st grade in 2015 (*n* = 4,291)			

	*N* or mean	% or [SD]	*N* or mean	% or [SD]
**Respondent** (answered ‘mother’)	3,884	90.5		
**Demographics of household**				
Families living in the same house	3.2	[1.1]		
The number of siblings	1.2	[0.9]		
Parental marital status				
Married	3,797	88.5		
Unmarried	54	1.3		
Divorced	278	6.5		
Bereaved	27	0.6		
Other/missing	135	3.2		
**Demographics of children**				
Sex (answered “boy”)	2,197	51.2		
Preschool facility				
Public nursery	1,122	26.1		
Private nursery	602	14.0		
Private kindergarten	2,377	55.4		
Other	155	3.6		
None	19	0.4		
Missing	16	0.4		
Difficult behavior (SDQ) of children				
Low need	3,014	70.2		
Some need	581	13.5		
High need	633	14.8		
Missing	63	1.5		
Pro-social behavior (SDQ) of children				
Low need	2,931	68.3		
Some need	718	16.7		
High need	583	13.6		
Missing	59	1.4		
**Parental characteristics**	Mother		Father	
Age, years	38.1	[5.1]	40.4	[5.8]
<30	222	5.2	86	2.0
30–34	764	17.8	461	10.7
35–39	1,455	33.9	1,147	26.7
40–44	1,310	30.5	1,318	30.7
≥45	418	9.7	836	19.5
Missing	122	2.8	443	10.3
Education				
Junior high school/high school	1,524	35.5	1,474	34.4
Technical/junior college/college dropout	1,763	41.1	846	19.7
Collage/graduate school	870	20.3	1,580	36.8
Other/missing	134	3.1	391	9.1
Employment				
Full-time	819	19.1	3,126	72.9
Part-time	1,599	37.3	108	2.5
Self-employed	208	4.8	580	13.5
Side work	80	1.9	1	0.0
Other	29	0.7	18	0.4
Not employed	1,461	34.0	42	1.0
Other/missing	95	2.2	416	9.7

SDQ, the Strengths and Difficulties Questionnaire; BMI, Body Mass Index.

In the A-CHILD study, poverty of children should be considered not only in terms of household economic hardship but also in the aspect of whole family environment. Therefore, we defined “households in living difficulty” as households that fell under any one of the following: (1) annual household income below 3 million JPY; (2) presence of material deprivation; and (3) experience of payment difficulty. For the following reasons, the threshold for economic difficulty was set to less than 3 million JPY per household. First, assuming maternal households receiving public assistance (for example, a 30s mother and a 1st-grade child in elementary school), the annual income will be equivalent to 2.72 million JPY when calculated based on welfare standards. In addition, there was little difference in the proportion of households with material deprivation and experience of payment difficulty between households with an annual income of 2 million JPY and households with 3 million JPY. To avoid missing the households in living difficulty, it seemed reasonable to consider that income groups with less than 3 million JPY lived in economic difficulty. In the questionnaire, we asked about things that respondents could not possess for economic reasons; not only home appliances for daily necessities and savings that can be dealt with when needed, but also places and goods which are considered necessary for child well-being (Table 4). We defined the existence of material deprivation when the respondent did not have at least one of the necessities. We also asked if they had any experience of being unable to pay for something in the past year (Table 4). In the first wave, the number of households with income of less than 3 million JPY was 489 (11.6%), the number of households with material deprivation was 670 (15.8%), and the number of households with payment difficulty was 389 (9.2%). The number of households with at least one of the above three aspects of poverty, that is, households in living difficulty, was 1,047 (24.5%) (Table 5). Among households receiving/received public aid, which was one of the indications of financial difficulty, the percentage of households in living difficulty was 84%. This high percentage indicates the validity of the definition of living difficulty.

**Table 4.** Definition categories of “Living difficulty” in A-CHILD study

**1) Household income below 3 million yen**		

**2) Existence of material deprivation**		
Books appropriate for children’s age	A vacuum	A phone (includes both landlines and mobiles)
Sports items, toys, or stuffed toys for children	Heaters/heating appliances	A bathtub per household
A place where children can study	An air-conditioner	A bed/mattress per person
A washing machine	A microwave	More than 50,000 yen in savings for emergencies
A rice cooker		

**3) Experience of payment difficulty**		
School field trips/extracurricular activities	Housing loans	Phone bills (includes both landlines and mobiles)
School textbooks	Electricity bills	Insurance fees for public pension, national health insurance, and/or public nursing care
School lunches	Gas bills	Bus or train fees for commuting
Rent	Water bills	

**Table 5.** The number of households with three aspects of poverty

	1st grade in 2015		2nd grade in 2016		4th grade in 2016		6th grade in 2016		8th grade in 2016	
	(*n* = 4,291)		(*n* = 4,358)		(*n* = 534)		(*n* = 530)		(*n* = 588)	
				
	*n*	%	*n*	%	*n*	%	*n*	%	*n*	%
Households in living difficulty	1,047	24.4	1,040	23.9	147	27.5	135	25.5	177	30.1
Annual household income (answered “less than JPY 3.0 million”)	489	11.4	483	11.1	62	11.6	66	12.5	89	15.1
Households with any material deprivation	670	15.6	691	15.9	99	18.5	83	15.7	117	19.9
Households with any payment difficulty	389	9.1	375	8.6	43	8.1	59	11.1	64	10.9

The details of each aspect of poverty are shown in supplementary materials.

### The second wave

Figure 3 shows the flow chart of subjects in the target grades of the second survey. Among the collected questionnaires, we removed the responses without consent for participation in the survey, those who did not answer the Study Attitude survey, those who did not have school health checkup data and those whose child refused to answer the child survey for the fourth, sixth, and eighth grades. We identified as valid responses 4,358 in second grade (81.4%), 534 in fourth grade (86.7%), 530 in sixth grade (85.1%), and 588 in eighth grade (77.9%).

The selected results of the questionnaire for the caregivers who participated in the second wave are shown in Table 6. Furthermore, the households of the second wave were also counted according to the aspects of poverty as the first wave (Table 4). Table 7 shows selected results of the questionnaires answered by children in the fourth, sixth, and eighth grades.

**Table 6.** Selected profiles of the participants for the 2nd wave of A-CHILD study

	2nd grade in 2016				4th grade in 2016				6th grade in 2016				8th grade in 2016			
	(*n* = 4,358)				(*n* = 534)				(*n* = 530)				(*n* = 588)			
			
	*N* or mean	% or [SD]	*N* or mean	% or [SD]	*N* or mean	% or [SD]	*N* or mean	% or [SD]	*N* or mean	% or [SD]	*N* or mean	% or [SD]	*N* or mean	% or [SD]	*N* or mean	% or [SD]
**Respondent**																
Mother	4,014	92.1			482	90.3			470	88.7			527	89.6		
Father	288	6.6			44	8.2			51	9.6			46	7.8		
Grandmother	11	0.3			4	0.7			4	0.8			3	0.5		
Grandfather	2	0.0			0	0.0			0	0.0			2	0.3		
Relatives/other	12	0.3			0	0.0			1	0.2			6	1.0		
Missing	31	0.7			4	0.7			4	0.8			4	0.7		
**Demographics of household**																
Families living in the same house	3.3	[1.1]			3.3	[1.1]			3.3	[1.2]			3.3	[1.2]		
The number of siblings	1.5	[0.7]			1.5	[0.7]			1.5	[0.7]			1.5	[0.8]		
Parental marital status																
Married					455	85.2			433	81.7			450	76.5		
Unmarried					8	1.5			3	0.6			11	1.9		
Divorced					48	9.0			73	13.8			89	15.1		
Bereaved					1	0.2			4	0.8			10	1.7		
Other/missing					22	4.1			17	3.3			28	4.7		
**Respondents’ psychosocial characteristics**																
Self-rated Health																
Good	1,447	33.2			148	27.7			163	30.8			164	27.9		
Somewhat good	1,466	33.6			195	36.5			185	34.9			180	30.6		
Normal	1,031	23.7			137	25.7			143	27.0			168	28.6		
Not very good	342	7.8			47	8.8			34	6.4			62	10.5		
Not good	39	0.9			2	0.4			1	0.2			8	1.4		
Missing	33	0.8			5	0.9			4	0.8			6	1.0		
**Demographics of children**																
Sex (answered “boy”)	2,228	51.1			279	52.2			238	44.9			288	49.0		
Preschool facility																
Public nursery					166	31.1			162	30.6			168	28.6		
Private nursery					62	11.6			60	11.3			48	8.2		
Private kindergarten					264	49.4			263	49.6			318	54.1		
Other					33	6.2			43	8.1			43	7.3		
None					7	1.3			2	0.4			9	1.5		
Missing					2	0.4							2	0.3		
Difficult behavior (SDQ)																
Low need	3,061	70.2			386	72.3			414	78.1			421	71.6		
Some need	551	12.6			64	12.0			54	10.2			75	12.8		
High need	714	16.4			80	15.0			59	11.1			84	14.3		
Missing	32	0.7			4	0.7			3	0.6			8	1.4		
Pro-social behavior (SDQ)																
Low need	2,940	67.5			359	67.2			364	68.7			348	59.2		
Some need	759	17.4			90	16.9			92	17.4			115	19.6		
High need	628	14.4			81	15.2			71	13.4			118	20.1		
Missing	31	0.7			4	0.7			3	0.6			7	1.2		
**Parental characteristics**	Mother		Father		Mother		Father		Mother		Father		Mother		Father	
Age, years					40.4	[4.8]	42.5	[5.9]	42.3	[5.0]	44.9	[5.9]	43.3	[4.8]	45.6	[6.0]
<30					3	0.6	2	0.4	4	0.8	2	0.4	0	0.0	0	0.0
30–34					60	11.2	37	6.9	31	5.8	13	2.5	24	4.1	12	2.0
35–39					138	25.8	96	18.0	106	20.0	54	10.2	83	14.1	51	8.7
40–44					210	39.3	189	35.4	194	36.6	157	29.6	218	37.1	142	24.1
≥45					92	17.2	151	28.3	177	33.4	226	42.6	233	39.6	262	44.6
Missing					31	5.8	59	11.0	18	3.4	78	14.7	30	5.1	121	20.6
Education																
Junior high school/high school					209	39.1	199	37.3	225	42.5	196	37.0	298	50.7	234	39.8
Technical/junior college/college dropout					215	40.3	106	19.9	217	40.9	90	17.0	208	35.4	120	20.4
Collage/graduate school					86	16.1	182	34.1	70	13.2	175	33.0	59	10.0	134	22.8
Other/missing					24	4.5	47	8.8	18	3.4	69	13.0	23	3.9	100	17.0
Employment																
Full-time	869	19.9	3,126	71.7	79	14.8	365	68.4	106	20.0	352	66.4	114	19.4	366	62.2
Part-time	1,772	40.7	85	2.0	266	49.8	19	3.6	271	51.1	10	1.9	296	50.3	9	1.5
Self-employed	215	4.9	613	14.1	30	5.6	91	17.0	30	5.7	83	15.7	33	5.6	87	14.8
Side work	83	1.9	4	0.1	10	1.9	5	0.9	5	0.9	0	0.0	3	0.5	0	0.0
Other	44	1.0	18	0.4	6	1.1	3	0.6	3	0.6	5	0.9	5	0.9	4	0.7
Not employed	1,271	29.2	25	0.6	124	23.2	51	9.6	103	19.4	7	1.3	114	19.4	8	1.4
Other/missing	104	2.4	487	11.2	19	3.6	0	0.0	12	2.3	73	13.8	23	3.9	114	19.4

SDQ, the Strengths and Difficulties Questionnaire.

**Table 7.** Selected profiles answered by children for the 2nd wave of A-CHILD study

	4th grade in 2016		6th grade in 2016		8th grade in 2016	
	(*n* = 534)		(*n* = 530)		(*n* = 588)	
	*N*	%	*N*	%	*N*	%
Dietary habit (answered “never”)						
Eating breakfast	6	1.1	4	0.8	8	1.4
Eating vegetables at breakfast	59	11.0	53	10.0	75	12.8
Dietary habit of sweets						
Eating sweets at on time	243	45.5	151	28.5	87	14.8
Never eating sweets	49	9.2	43	8.1	66	11.2
Eating sweets anytime	235	44	336	63.4	435	74.0
Missing	7	1.3	0	0.0	0	0.0
Frequency of physical activity						
More than 2 times/week	215	40.3	195	36.8	331	56.3
1–2 times/week	229	42.9	207	39.1	69	11.7
1–3 times/month	53	9.9	81	15.3	76	12.9
Never	31	5.8	43	8.1	105	17.9
Missing	6	1.1	4	0.8	7	1.2
Time spent on mobile devices (answered “≥4 hours”)	11	2.0	35	6.7	99	16.8
The number of books that one has read within one month (answered “hasn’t read any”)	79	14.8	101	19.1	167	28.4
Toothbrushing habit (answered “both morning and night”)	414	77.5	439	82.8	469	79.8
Daily time of watching TV or video						
Never/rarely	54	10.1	24	4.5	24	4.1
About 0.5–2.5 hours	369	69.1	361	68.2	365	62.0
≥3 hours	95	17.8	131	24.7	171	29.0
Missing	16	3.0	14	2.6	28	4.8

The results of physical measurement and dental checkup in school health checkup are shown in Table 8. Among the target children of in the eighth grade in the second survey in 2016, 502 (85.4%) underwent additional blood test and a measurement of blood pressure. The averages of the results are also shown in Table 8.

**Table 8.** Selected profiles of the school health checkup in the second survey of A-CHILD survey in 2016

	2nd grade in 2016		4th grade in 2016		6th grade in 2016		8th grade in 2016	
	(*n* = 4,358)		(*n* = 534)		(*n* = 530)		(*n* = 588)	
			
	*N* or mean	% or [SD]	*N* or mean	% or [SD]	*N* or mean	% or [SD]	*N* or mean	% or [SD]
**BMI**	16.0	[1.9]	16.9	[2.7]	18.1	[3.2]	19.6	[3.0]
**Deciduous tooth decayed or filled (dft)**								
None/not applicable	2,391	54.9	281	52.6	403	76.0	548	93.2
1–2 teeth	819	18.8	140	26.2	96	18.1	20	3.4
3–4 teeth	442	10.1	58	10.9	21	4.0	1	0.2
5 and more teeth	662	15.2	50	9.4	6	1.1	0	0.0
Missing	44	1.0	5	0.9	4	0.8	19	3.2
**Permanent tooth decayed, missing or filled (DMFT)**								
None/not applicable	4,029	92.5	461	86.3	419	79.1	420	71.4
1–2 teeth	239	5.5	54	10.1	84	15.8	102	17.3
3–4 teeth	46	1.1	14	2.6	17	3.2	32	5.4
5 and more teeth	0	0.0	0	0.0	6	1.1	15	2.6
Missing	44	1.0	5	0.9	4	0.8	19	3.2
**Blood test (parts of 8th grade students only, *n* = 502)**								
Systolic blood pressure (mm Hg)							111.2	[9.4]
Diastolic blood pressure (mm Hg)							60.3	[6.9]
Total cholesterol (mg/dL)							166.4	[27.5]
HDL-cholesterol (mg/dL)							63.1	[12.2]
LDL-cholesterol (mg/dL)							91.5	[23.3]
Red blood cells count (10,000/µL)							489.8	[39.4]
Hemoglobin (g/dL)							14.0	[1.1]
Hematocrit (%)							42.3	[3.0]
Hemoglobin A1c (%)							5.3	[0.2]

Missing rates for some questions were very low (eg, 0.1% for child sex in first-graders in 2015). On the other hand, questions related to the finance in households, such as household income (5.5% in 2015) and receipt of some kind of public aid (3.5–14.8% in 2015), tended to be higher missing rate (results not shown in tables). We assume those missing values would not have occurred at random; that is, systematic differences should remain between the missing values and the observed values. We did not address any adjustments for those missing and maintained them in the data, because it should be left to the researchers how to handle incomplete data, in accordance with each research question.

## STRENGTHS AND LIMITATIONS

The Japanese act to accelerate the development of policies for disadvantaged children declares that children in poverty should grow up in a desirable environment in terms of education, living conditions, working conditions, and economic perspectives, so that they are not adversely affected by the environment in which they were born and raised in. The principle of this anti-child-poverty act in Japan aligns with the recent public health goal that we should tackle the social determinants of health to reduce the health gap, which is outlined in the initiative policy by World Health Organization.^29^

The A-CHILD study is one of the most comprehensive life-course studies of Japanese children and parents. It covers socio-economic factors and medical aspects. There were few Japanese data with both detailed indicators of each child lifestyle and objective health data using doctor’s measurements, dental diagnoses, and blood test values. The subjects who have been longitudinally surveyed since 2015 are all first-grade children of public elementary schools and their family in Adachi City. Additionally, biennial surveys targeting elementary and junior high school grades children have been conducted at schools selected in consideration of regional and social backgrounds in Adachi City since 2016. This research design allows us to examine the factors and interactions that determine health and its disparities in a child’s growth process at the individual, household and school, and regional level, by adopting an appropriate analytic strategy, such as multi-level analysis.

However, it should also be noted that the A-CHILD study does have its limitations. First, the study was conducted in Adachi City, which is one of the 23 special wards in Tokyo, and the targeted schools for biennial observational surveys of children in the fourth grade and higher make up only a portion of the public schools in the city. In addition, while the data of the “child lifestyle-related health checkup” with blood test and blood pressure measurements are valuable, only applicants in eighth grade underwent this checkup. For these reasons, the generalizability of the result from the A-CHILD study may be limited. Our target population is the children who lived in Adachi City; however, we were not sure whether the results from those data of participants in public school and their parents would represent all targeted children. In order to proximate our results to a generalizable estimate, researchers will need other data of a representative sample, including the children such as those who lived in Adachi City but went to a private school in another city. Second, although academic ability is an important factor in determining the future of children, such as educational achievement and employment, the data of A-CHILD do not include children’s academic measurement. Going forward, we expect to link objective measures of academic ability, such as annual academic assessment conducted at schools, to the A-CHILD data. Third, most of the respondents of the caregiver’s questionnaire were mothers of children, so information on the father might not be accurate. Fourth, the A-CHILD’s essential purpose is to understand the actual conditions of children and families living in difficulty, but it was unclear whether all parents and children in disadvantaged situations had been able to participate in the survey. Although the response rates for A-CHILD were high enough for any grade, some questionnaires remained unanswered. For example, if the parents were rarely at home, or were seriously sick, or if familial relationships were not working, the questionnaire might not be answered by their caregivers. Also, caregivers who were not native Japanese may find it difficult to answer because the questionnaire was in Japanese. In A-CHILD, we indicated contact information not only in Japanese but also English, Chinese, Korean, and Tagalog, but there was no actual inquiry. Lastly, given that drop-outs from the cohort study would differ in several ways from the respondents, any conclusions drawn from the study may be biased. Previous studies suggested that dropout from the cohort study was not at random but tended to be higher among lower socio-economic populations.^30^^–^^32^ Therefore, any additional treatment for non-response in the follow-up, such as inverse probability weighting or multiple imputation,^33^^–^^39^ could be a benefit for the researcher using the longitudinal data of A-CHILD for handling potential non-random dropouts.

In spite of these limitations, almost all local municipalities in Japan have now begun to tackle the issue of child poverty, with the results obtained in Adachi City being very meaningful and helpful for these municipalities. In the future, it is necessary to clarify whether experiences of living difficulty can influence physical and psychological development, behaviors, and the health status of children as they get older. For that purpose, we plan to continue to follow the children participating in the A-CHILD longitudinal survey until at least the eighth grade and beyond. With these advantages, the A-CHILD will continue to contribute important information for the development of policies to tackle the issue of child poverty. Such information includes which aspects of poverty are the most harmful in terms of a child’s physical and mental development and which types of experiences with people in school or community can foster the ability and growth of children. By obtaining such information, we postulate the pathway to reduce health disparities among children in the future will be clearer.

## APPENDIX A. SUPPLEMENTARY DATA

eAppendix 1. Supplemental methods
