# Supplementary material for: Adachi Child Health Impact of Living Difficulty (A-CHILD) Study: Research Protocol and Profiles of Participants
Source: J Epidemiol. 2021 Jan 5;31(1):77–89. doi: 10.2188/jea.JE20190177 (PMC7738641; doi:10.2188/jea.JE20190177)
Supplement: Supplementary file 1 [file je-31-077-s001.pdf]

eAppendix 1. Supplemental methods

Title

Adachi Child Health Impact of Living Difficulty (A-CHILD) Study: Research Protocol and Profiles of Participants

List

Respondents' mental health

Difficult behavior and prosocial behavior in children

Resilience in children

Physical measurement and dental checkup

Measurement items of "child lifestyle-related health checkup" among 8th-grade students

Respondents' mental health

Respondent's mental health was assessed using a translated and validated version in Japanese of the K6 scale,[1, 2] which employs a 5-point response options from 0 (= none of the time) to 4 (= all of the time) with a scale score range of 0–24. Respondents were classified into two groups: those with and without psychological distress according to recommended cut-off points.

Difficult behavior and prosocial behavior in children

Regarding behavioral problems and prosocial behavior in children, caregivers were asked to answer the Strengths and Difficulties Questionnaire (SDQ)[3] using a validated Japanese

version of the questionnaire.[4] The subscales of SDQ are emotional symptoms, conduct problems, hyperactivity-inattention problems, peer relationship problems, and prosocial behavior. The total difficulties score is calculated by summing up the score of four subscales of behavioral difficulties, except for scales of prosocial behavior.

Resilience in children

Child resilience was assessed using the Children's Resilient Coping Scale (CRCS) in the questionnaire, which consists of eight items developed by Japanese experts to suit the Japanese context based on previous studies related to resilience and coping skills.[5-7] The eight items of the CRCS were: (1) speaks positively about their future; (2) tries their best; (3) able to take teasing or mean comments well; (4) knows how to greet others properly; (5) able to get ready for school, study, and do his/her chores without directions; (6) seeks appropriate advice when necessary; (7) able to give up on things they want or do things that they do not like to do for better future outcomes; and (8) able to ask questions to learn about what they did not understand. For these items, caregivers rated child resilience/coping behaviors using a scale of 0 (never) to 4 (very frequently). A higher total score denoted a higher level of resilience and coping skill.

Physical measurement and dental checkup

School teachers assessed the children’s height and weight. Height was measured to the nearest 0.1 cm using a portable stadiometer, and weight to the nearest 0.1 kg on a digital scale wearing light clothing without shoes. Dentists conduct dental checkups. According to the guideline, dentists examine children’s every tooth in school using a dental mirror and ball-ended probe or the Community Periodontal Index (CPI) probe under sufficient

lighting. Dental units or radiography were not used. White lesions were not counted as dental caries.[8]

Measurement items of "child lifestyle-related health checkup" among eighth-grade students

As for blood test, total cholesterol (mg/dL), HDL-cholesterol (mg/dL), LDL-cholesterol (mg/dL), red blood cells count (10,000/µL), hemoglobin (g/dL), hematocrit (%), and hemoglobin A1c (%) were measured from blood taken from the arm without fasting at a laboratory. Glycosuria was also assessed from morning urine. Blood pressure at resting was assessed by nurses.

eTable 1. The number of households with three aspects of poverty

	1st grade in 2015 (n = 4,291)		2nd grade in 2016 (n = 4,358)		4th grade in 2016 (n = 534)		6th grade in 2016 (n = 530)		8th grade in 2016 (n = 588)	
	n	%	n	%	n	%	n	%	n	%
**Households in living difficulty**	1,047	24.4	1,040	23.9	147	27.5	135	25.5	177	30.1
**Annual household income (million yen)**										
<3.0	489	11.4	483	11.1	62	11.6	66	12.5	89	15.1
3.0–5.9	1,718	40.0	1,780	40.8	207	38.8	189	35.7	193	32.8
6.0–9.9	1,284	29.9	1,332	30.6	167	31.3	175	33.0	190	32.3
≥10.0	360	8.4	414	9.5	54	10.1	63	11.9	49	8.3
Unknown	440	10.3	349	8.0	44	8.2	37	7.0	67	11.4
**Households with any material deprivation**	670	15.6	691	15.9	99	18.5	83	15.7	117	19.9
Books appropriate for child’s age	101	2.4	99	2.3	22	4.1	14	2.6	24	4.1
Sports items, toys, or stuffed toys for children	57	1.3	75	1.7	13	2.4	10	1.9	13	2.2
A place where my child can study	142	3.3	132	3.0	26	4.9	12	2.3	30	5.1
A washing machine	8	0.2	26	0.6	3	0.6	1	0.2	6	1.0
A rice cooker	14	0.3	23	0.5	3	0.6	1	0.2	6	1.0
A vacuum	11	0.3	33	0.8	5	0.9	2	0.4	7	1.2
Heaters/heating appliances	25	0.6	30	0.7	3	0.6	1	0.2	6	1.0
An air-conditioner	24	0.6	31	0.7	5	0.9	0	0.0	7	1.2
A microwave	15	0.3	33	0.8	5	0.9	3	0.6	7	1.2
A phone (includes both landlines and mobiles)	61	1.4	68	1.6	8	1.5	6	1.1	13	2.2

A bathtub per household	15	0.3	23	0.5	3	0.6	1	0.2	7	1.2
A bed/mattress per person	130	3.0	151	3.5	18	3.4	11	2.1	18	3.1
More than 50,000 yen in savings for emergencies	498	11.6	522	12.0	67	12.5	66	12.5	83	14.1
**Households with any payment difficulty**	389	9.1	375	8.6	43	8.1	59	11.1	64	10.9
School field trips/extracurricular activities	37	0.9	16	0.4	1	0.2	0	0.0	4	0.7
School textbooks	14	0.3	38	0.9	5	0.9	6	1.1	13	2.2
School lunches	64	1.5	88	2.0	8	1.5	11	2.1	14	2.4
Rent	83	1.9	61	1.4	5	0.9	10	1.9	5	0.9
Housing Loans	39	0.9	40	0.9	5	0.9	3	0.6	7	1.2
Electricity bills	98	2.3	65	1.5	8	1.5	4	0.8	12	2.0
Gas bills	91	2.1	52	1.2	9	1.7	3	0.6	11	1.9
Water bills	87	2.0	60	1.4	11	2.1	9	1.7	10	1.7
Phone bills (includes both landlines and mobiles)	88	2.1	72	1.7	10	1.9	13	2.5	17	2.9
Insurance fees for public pension, national health insurance, and/or public nursing care	279	6.5	254	5.8	27	5.1	38	7.2	32	5.4
Bus or train fees for commuting	19	0.4	9	0.2	2	0.4	1	0.2	5	0.9

REFERENCES

1. Kessler RC, Andrews G, Colpe LJ, Hiripi E, Mroczek DK, Normand S-LT, et al. Short screening scales to monitor population prevalences and trends in non-specific psychological distress. Psychol Med. 2002;32:959-76.
2. Furukawa TA, Kawakami N, Saitoh M, Ono Y, Nakane Y, Nakamura Y, et al. The performance of the Japanese version of the K6 and K10 in the World Mental Health Survey Japan. Int J Methods Psychiatr Res. 2008;17:152-8.
3. Goodman R. The strengths and difficulties questionnaire: A research note. J Child Psychol Psychiatry. 1997;38:581-6.
4. Matsuishi T, Nagano M, Araki Y, Tanaka Y, Iwasaki M, Yamashita Y, et al. Scale properties of the Japanese version of the Strengths and Difficulties Questionnaire (SDQ): a study of infant and school children in community samples. Brain Dev. 2008;30:410-5.
5. Lazarus RS. COPING THEORY AND RESEARCH - PAST, PRESENT, AND FUTURE. Psychosom Med. 1993;55:234-47.
6. Compas BE, Connor-Smith JK, Saltzman H, Thomsen AH, Wadsworth ME. Coping with stress during childhood and adolescence: Problems, progress, and potential in theory and research. Psychol Bull. 2001;127:87-127.
7. Compas BE, Jaser SS, Dunbar JP, Watson KH, Bettis AH, Gruhn MA, et al. Coping and emotion regulation from childhood to early adulthood: Points of convergence and divergence. Aust J Psychol. 2014;66:71-81.
8. Children’s Health Diagnostic Manual (Revised edition). Tokyo. Japanese Society of School Health; 2006.
